# Evaluation and Clinical Validity of a New Questionnaire for Mikulicz's Disease

**DOI:** 10.1155/2012/283459

**Published:** 2012-05-08

**Authors:** Motohisa Yamamoto, Hiroki Takahashi, Keisuke Ishigami, Hidetaka Yajima, Yui Shimizu, Tetsuya Tabeya, Mikiko Matsui, Chisako Suzuki, Yasuyoshi Naishiro, Hiroyuki Yamamoto, Kohzoh Imai, Yasuhisa Shinomura

**Affiliations:** ^1^First Department of Internal Medicine, School of Medicine, Sapporo Medical University, Hokkaido 0608543, Japan; ^2^Advanced Clinical Research Center, The Institute of Medical Science, The University of Tokyo, Tokyo 1088639, Japan

## Abstract

*Objectives*. The characteristic features of Mikulicz's disease (MD) are diffuse enlargement of the lacrimal and submandibular glands, elevated levels of serum immunoglobulin (Ig)G4, and abundant infiltration of IgG4-positive plasmacytes into both glands. No disease index is available to properly evaluate MD, so we developed a functional assessment of MD, the Mikulicz's disease activity questionnaire (MAQ), and evaluated its clinical efficacy. *Methods*. We selected 18 patients who were either being treated for MD or who had presented with recurrence. The patients completed a self-assessment and were scored according to the MAQ sheet during each visit between December 2009 and August 2011. Assessment items were in regard to increases or decreases in lacrimal and salivary gland enlargement and severity of sicca symptoms. *Results*. On the first visits, MAQ scores were high, but scores decreased rapidly as treatment progressed. When doses of glucocorticoid were reduced, some patients showed increased scores. Dry-symptom scores increased initially. MAQ scores for patients with recurrent MD gradually increased over several months before relapse. However, some patients displayed no elevation in MAQ scores due to relapses at other sites. *Conclusion*. MAQ score can be used to quantify flares and treatment response and is useful for functional assessment of MD.

## 1. Background and Purpose

 Mikulicz's disease (MD) is a chronic inflammatory disease characterized by diffuse enlargement of the lacrimal and submandibular glands, elevated levels of serum immunoglobulin (Ig)G4, and abundant infiltration of IgG4-positive plasmacytes and fibrosis in both glands. MD is considered as an IgG4-related disease (IgG4-RD) with aspects of systemic disorders [1]. Several epidemiological studies have shown that thousands of patients in Japan have MD [2]. The pathogenesis of IgG4-RD involves a gradual shift from an inflammatory stage to a fibrotic stage. Early intervention with appropriate therapy is necessary to avoid irreversible organ dysfunction. One of the features of MD is a high relapse rate following reductions in glucocorticoid treatment, as this steroid is known to be useful in achieving clinical remission [3]. 

 As a representative chronic disorder, treatment of rheumatoid arthritis (RA) requires precise clinical evaluation and review of the course of treatment [4]. Similar principles would apply to MD, but no clinical index has been available to properly evaluate this pathology. The health assessment questionnaire (HAQ) was developed as a comprehensive measure of outcomes in patients with a wide variety of rheumatic diseases [5], but does not reflect the condition of MD particularly well because MD is a local condition affecting only the lacrimal and salivary glands. A system for assessing disease activity and functional impairment in patients with MD is thus needed, and we therefore developed a functional evaluation system, the “Mikulicz's disease activity questionnaire” (MAQ), and have applied this in daily practice since December 2009. The present study analyzed the clinical efficacy of MAQ in patients with MD. 

## 2. Methods

 We conducted a followup study of 18 MD patients from December 2009 to August 2011. Diagnoses were made according to the diagnostic criteria for IgG4-related MD proposed by the Japanese Society for Sjögren's syndrome in 2008 (Figure 4) [6] and by the pathological evaluation of enlarged submandibular glands. Study subjects included patients who had started treatment and patients who presented with relapses. Patients under 20 years old were excluded. At the hospital and related institutes, patients assessed the severity of their own lacrimal and salivary gland swelling and recorded the scores on the MAQ. Levels of serum IgG4 were also measured continuously. We analyzed the serial changes in MAQ score with treatment. 

**Figure 1:**
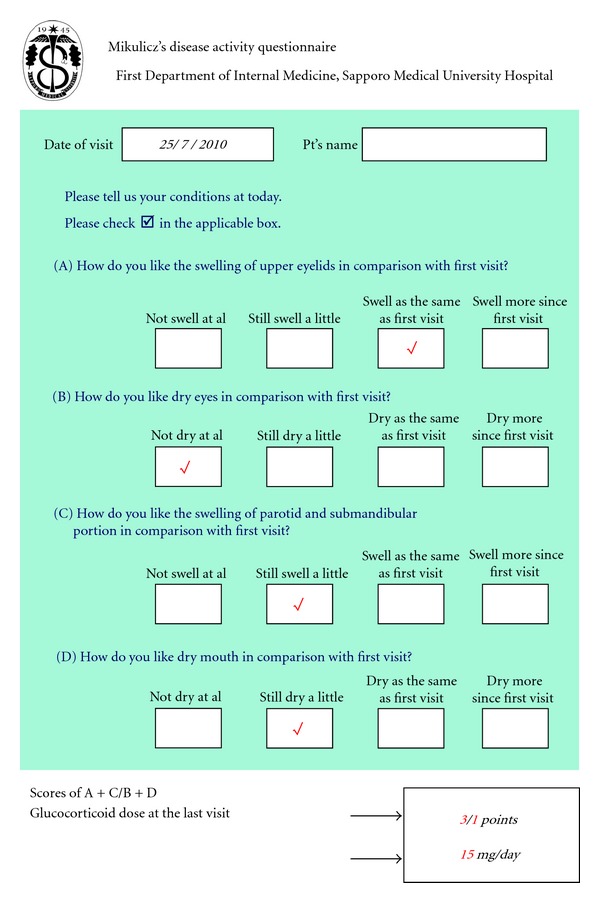
The English version of the Mikulicz's disease activity questionnaire (MAQ). An example of a completed MAQ as used in the clinic during routine visits by patients.

 The MAQ score sheet (Figure 1) comprises four questions to assess the degrees to which the lacrimal and salivary glands were enlarged and the occurrence and severity of sicca symptoms. Patients checked the boxes that best corresponded to current symptoms: disappearance of symptoms (0 points); slight improvement of symptoms (1 point); unchanged symptoms (2 points); worsening of symptoms (3 points). MAQ scores were determined as the total of the swelling-evaluation points and dryness-evaluation points, allowing us to compare the conditions of patients compared with the first visit. We also recorded the amount of prescribed glucocorticoid at each visit. No patients were given any guidance including leading questions in this assessment. If marked differences were seen in assessment results between patients and doctors, we asked the doctors to record comments in the MAQ sheets. In MD, relapse was defined as reenlargement of the lacrimal and salivary glands on physical and image findings. In other organs, MD relapse was defined as swelling of organs on systemic enhanced CT examined periodically. However, for renal lesions, we considered the appearance of contrast defects in the renal parenchyma as indicative of flare-up.

Treatment was performed as follows. For patients with failure of organs other than the lacrimal and salivary glands, we initially prescribed 0.8 mg/kg/day of prednisolone (PSL) for 1 month and then reduced the amount by 10% every 2 weeks. For those patients without organ failure, we initially prescribed 0.6 mg/kg/day of PSL [6]. After the dose was decreased to <10 mg/day, we continued to administer a maintenance dose for 6 months. After 6 months, the doctors reduced the amount of steroid if the patient was in clinical remission. If a relapse occurred, the dose of glucocorticoid was increased. In our analysis, clinical remission was defined as no observation of lacrimal or salivary gland enlargement over a 3-month period as determined by physical and imaging findings. Recurrence was defined as the necessity for treatment intervention due to swelling of the glands or detection of other organ dysfunction. 

## 3. Results

### 3.1. Patient Profiles

 The 41 participants in the followup study comprised 17 men and 24 women. Mean (±standard deviation) age at MD onset was 56.59 ± 11.90 years, and mean age at diagnosis was 58.44 ± 11.52 years. Mean duration of followup from the first visit was 4.00 ± 2.36 years. Seven patients (17.1%) started treatment with glucocorticoid, and 11 patients (26.8%) presented with recurrence during the observation period. As of December 2009, 23 cases had been prescribed with steroid and showed no flare-up during the study period (Table 1).

### 3.2. Analysis of Patients Starting Treatment

**Figure 2:**
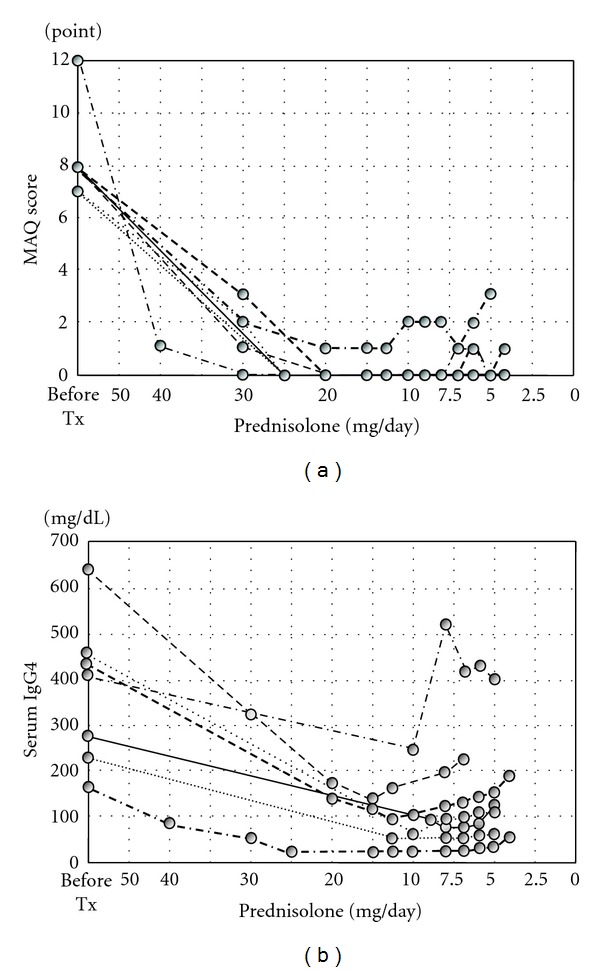
Serial changes in MAQ scores and serum IgG4 levels in patients who started treatment during the observation period. (a) MAQ scores decreased rapidly after starting treatment. Some patients showed initial sicca symptoms, and scores rose when the steroid dose was reduced to 8–10 mg/day. (b) Serum IgG4 levels decreased after initiation of treatment. Some patients showed reelevation of IgG4 levels prior to MAQ scores at <15 mg/day of PSL. Virtually no cases showed any elevation of serum IgG4 at the dosage of 5 mg/day.

 The 7 patients who began treatment during the observation period showed high MAQ scores of 8.3 ± 1.7 at the first visit. Starting steroid doses were as follows: 40 mg/day in 1 patient; 30 mg/day in 5 patients; 25 mg/day in 1 patient. Doctors continued to prescribe PSL at 4 to 7 mg/day at last visit. As all patients progressed with treatment, MAQ scores including dryness scores rapidly decreased; scores were <1 in all cases when the dose of PSL was reduced to 20 mg/day. Some cases initially presented with sicca symptoms and subsequently showed an increase in questionnaire scores at 8 to 10 mg/day (Figure 2(a)). In untreated patients, however, mean serum level of IgG4 was 368.71 ± 162.28 mg/dL. Patient levels of IgG4 also decreased after the initiation of treatment, but at <15 mg/day of PSL, IgG4 levels in some patients reelevated in advance of reelevations in MAQ scores. Almost all patients showed an elevation of serum IgG4 at the dose point of 5 mg/day (Figure 2(b)). In this study, no cases were seen in which assessments differed markedly between patients and doctors.

### 3.3. Analysis of Patients Presenting with Relapse

**Figure 3:**
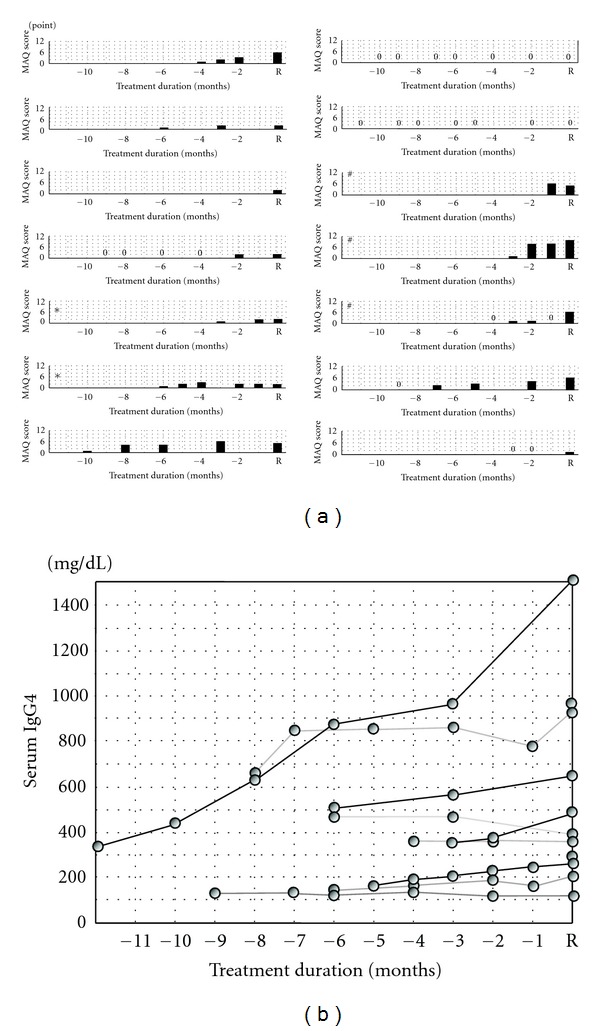
Serial changes in MAQ scores and serum IgG4 levels in patients with relapse (R). (a) Serial changes in MAQ scores. In many cases, MAQ scores had gradually elevated 1 to 6 months before relapse. ∗ and # in the bar graphs denote the same cases. Case ∗ showed two flares, while case # experienced relapse three times. (b) Serial changes in serum IgG4 levels which had elevated several months before relapse.

**Figure 4:**
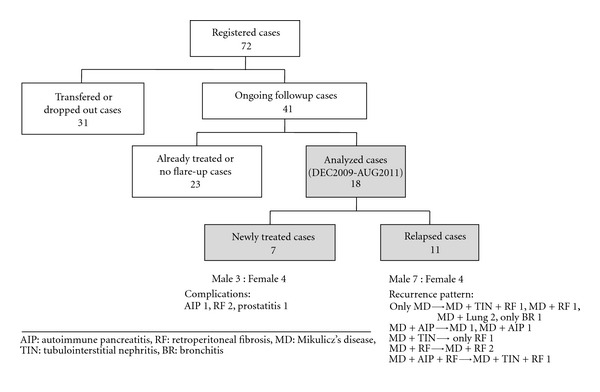
Diagnostic criteria for IgG4-related MD (as proposed by the Japanese Society for Sjögren's syndrome in 2008).

 Among the 11 patients who presented with relapses during observation, 2 experienced more than one relapse. The steroid dose for these patients at the time of relapse was 0 to 16 mg/day, and the mean dose was 6.86 ± 4.54 mg/day. Most of these patients received an additional amount of steroid, but patients with repeated past recurrences received immunosuppressants such as mizoribine or rituximab. Mean MAQ scores at first relapse were 2.7 ± 2.1, with scores of 0 in two cases. Many cases showed a gradual elevation in MAQ scores at 1 to 6 months before relapse (Figure 3(a)). This tendency toward elevation was marked in dryness scores. In two patients who presented with more than one relapse, MAQ scores showed an unstable transition after the PSL dose was increased. We also found that these patients showed elevated levels of serum IgG4 several months before relapse (Figure 3(b)). Levels of serum IgG4 decreased immediately after an increase in steroid dose, but reelevation of serum IgG4 was observed with a reduction of glucocorticoid (data not shown). As with patients starting treatment, no cases showed marked differences in assessments between patients and doctors.

## 4. Discussions

 MD is a chronic inflammatory disorder and can also be termed IgG4-related dacryoadenitis and sialadenitis. Disease activity indices are needed in the assessment of chronic diseases. Glucocorticoid treatment is now uniformly performed for MD, but the disease is known to be likely to relapse with reductions in steroid dose. In our data (SMART: Sapporo Medical University and related institutes database for investigation and best treatments of IgG4-related disease), patients withdrawn from steroid presented with mild symptoms (data not shown). We may thus be able to select the dose of glucocorticoid or nonsteroidal treatment, including biologic agents, depending on disease activity during therapy for MD. Recurrence of MD is currently often diagnosed based on physical and imaging findings. On the other hand, risk factors for relapse remain unclear, and no studies have reported analyses of flare-up signs in MD. We therefore developed the MAQ as a clinical evaluation scoring system for MD and analyzed the clinical application of this questionnaire. 

 The lacrimal and salivary glands are involved in MD. Our database revealed that the chief complaints in most patients are glandular enlargement and impaired secretion. Other complaints, including pain, are rarely observed. We therefore asked patients to assess swelling and dryness in the lacrimal and salivary glands. The degrees of subjective symptoms were set at four levels compared to the first visit: disappearance of symptoms, slight improvement, no change, or progression. This was in consideration of patients, to avoid them wondering which option they should check. This approach to assessment was well received by patients and did not interfere with practice.

 As expected, results showed high MAQ scores before treatment and decreased scores after glucocorticoid administration. Many patients showed improvements of more than 5 points, with improvements in both swelling and dryness. We have previously described the efficacy of glucocorticoids in short-term treatment [3]. This analysis demonstrated that the MAQ accurately reflects such results. However, some patients still presented with sicca symptoms after undergoing steroid treatment. MD is not considered prone to destruction of the glands [7], but progressive fibrosis can irreversibly reduce glandular function. Different factors can also affect sicca symptoms, including mental condition, diabetes mellitus, and concomitant medications. Ideally, each individual factor should be assessed in terms of impacts on dryness, but this is obviously impractical to achieve. The MAQ is expected to adequately evaluate the changing condition of the same patients if no other factors change.

With respect to relapse, the elevation of dryness scores preceded that of swelling scores in most cases. The origin of glandular enlargement in MD remains unclear, but glandular impairment may occur before swelling in MD. The present analysis did not include any missed cases with recurrence. MAQ scores appeared to accurately reflect relapses in MD. However, two patients presented with relapse in other organs despite MAQ scores of 0. One case exhibited relapse from MD alone to bronchitis, and another showed relapse from MD with IgG4-related tubulointerstitial nephritis to retroperitoneal fibrosis alone. In the other cases of flare-up, five patients presented with the involvement of other organs. In all, new lesions in other organs at the time of relapse were revealed in 7 of the 11 cases. This result offers a good reminder that MD is a systemic disease. To address this issue, developing indices for each organ and then combining them is one option, but development of an integrated assessment may be a better approach. The activity index for IgG4-RD requires one such integrated assessment. 

The present analysis also examined the association between MAQ scores and serum level of IgG4. Levels of serum IgG4 elevated 3 to 6 months before clinical identification of recurrence. MAQ scores tended to increase later than levels of serum IgG4. Cases presenting with other organ involvements despite an MAQ score of 0 also showed elevated serum IgG4 levels. Tabata pointed out the possibility that concentrations of serum IgG4 reflect IgG4-RD disease activity [8]. Fluctuations in serum IgG4 levels would thus also be important in assessing the disease activity of IgG4-RD. Comparing degrees of elevation of serum IgG4 in flare-ups among patients is difficult, given the wide variations between individual cases. We therefore did not examine correlations between the degree of IgG4 elevation and changes in MAQ score in the present study.

 Clinical research in the present study was designed to maximize the number of cases within a limited period, but this study remains ongoing. As a result, we are hopeful that we will encounter novel findings using the integrated data over the longer term. We would like to perform a multicenter study and ascertain the utility of MAQ scores on a large scale in the future. The MAQ appears to offer a useful assessment of disease activity in MD and may help to catalyze the development of a disease activity index for IgG4-RD.

## Figures and Tables

**Table 1:** Characteristics of analyzed patients with Mikulicz's disease.

(1) Persistent (>3 months), symmetrical swelling of the lacrimal, parotid, and submandibular glands, involving at least two pairs.	
(2) Serologically high levels of immunoglobulin (lg)G4 (≥13.5 mg/L).	
(3) Marked lgG4-positive plasmacyte infiltration (≥50% lgG4-positive/lgG-positive cells in five high power fields) into lacrimal and salivary gland tissues.	

In terms of diagnosis, lgG4-related Mikulicz's disease is defined as satisfying item 1 and either item 2 and/or item 3. This form of systemic lgG4-related disease often accompanies multiple organ lesions. Sarcoidosis, Castleman's disease, Wegener's granulomatosis, and malignant lymphoma need to be considered as differential diagnoses.
